# Classification of Electroencephalogram Signal for Developing Brain-Computer Interface Using Bioinspired Machine Learning Approach

**DOI:** 10.1155/2022/4487254

**Published:** 2022-02-25

**Authors:** M. Thilagaraj, S. Ramkumar, N. Arunkumar, A. Durgadevi, K. Karthikeyan, S. Hariharasitaraman, M. Pallikonda Rajasekaran, Petchinathan Govindan

**Affiliations:** ^1^Department of Electronics and Instrumentation Engineering, Karpagam College of Engineering, Coimbatore, India; ^2^School of Computing, Kalasalingam Academy of Research and Education, Virudhunagar, India; ^3^Department of Biomedical Engineering, Rathinam Technical Campus, Coimbatore, India; ^4^Department of Electrical and Electronics Engineering, K. Ramakrishnan College of Engineering, Trichy, India; ^5^Department of Electrical and Electronics Engineering, Ramco Institute of Technology, Rajapalayam, India; ^6^School of Computer Science and Engineering, VIT Bhopal, Bhopal, Madhya Pradesh, India; ^7^Department of Electronics and Communication Engineering, Kalasalingam Academy of Research and Education, Virudhunagar, India; ^8^Department of Electrical and Electronics Technology, Ethiopian Technical University, Addis Ababa, Ethiopia

## Abstract

Transforming human intentions into patterns to direct the devices connected externally without any body movements is called Brain-Computer Interface (BCI). It is specially designed for rehabilitation patients to overcome their disabilities. Electroencephalogram (EEG) signal is one of the famous tools to operate such devices. In this study, we planned to conduct our research with twenty subjects from different age groups from 20 to 28 and 29 to 40 using three-electrode systems to analyze the performance for developing a mobile robot for navigation using band power features and neural network architecture trained with a bioinspired algorithm. From the experiment, we recognized that the maximum classification performance was 94.66% for the young group and the minimum classification performance was 94.18% for the adult group. We conducted a recognizing accuracy test for the two contrasting age groups to interpret the individual performances. The study proved that the recognition accuracy was maximum for the young group and minimum for the adult group. Through the graphical user interface, we conducted an online test for the young and adult groups. From the online test, the same young-aged people performed highly and actively with an average accuracy of 94.00% compared with the adult people whose performance was 92.00%. From this experiment, we concluded that, due to the age factor, the signal generated by the subjects decreased slightly.

## 1. Introduction

Pseudocoma is also known as the locked-in syndrome (LIS), which affects people and makes them cannot move or communicate verbally. People with LIS are unable to communicate with others due to complete paralysis. LIS affects individuals cognitively and emotionally and makes them unable to speak and move. LIS affects and damages the brainstem part called the pons. Pons is responsible for sharing the neural communication between others parts like cerebellum, cerebrum, and spinal cord. It affects both males and females equally from children to aged people, but most of the time, it causes severe damage to adult people. LIS stops neural communication from the brain to the spinal cord and other remaining master parts of the body, so the bodies of the affected people are not working properly. So, people are able to communicate only through some of the coded messages by giving signals without moving the body parts [1–3].

We are living in a modern and scientific world. Today several technologies are available for us to make communication in the absence of biochannels. So there is a need for rehabilitative devices for people with LIS using EEG-supported BCI. The EEG-based rehabilitative device was simple, accurate, and low cost. Due to this reason, most of the locked-in state people select the EEG-based rehabilitative device for communication. The brain activities captured from the EEG were converted into actions through the interface, which is called BCI. The technique of measuring brain functions and trying to identify the connection between certain brain activities through definite mental or other activities is called electroencephalogram (EEG). It is a tool to calculate cognitive neuropsychology and neuroscience. EEG simulates brain activities in the form of signals by inserting the electrodes on the scalp of the individual person, and most of the time, EEG was applied in the form of a noninvasive technique [4–8].

Most of the BCIs convert human intentions into control signals. These signals were used to control the machines in the lack of a normal channel. Some of the important BCIs that help paralyzed people to behave like normal people include mouse controller [9–11], speech synthesizer [12–15], robotic arm [16], hand controller [17], keyboard and mouse controller [18], facial expression detector [19], game controller [20], and mobile phone controller [21]. In our study, we planned to conduct a comparative study between two age groups, 20–28 and 29–40, to inspect the performance in offline and online modes using a recurrent neural network trained with a metaheuristic algorithm. The performance was compared with traditional methods to analyze the best performance for developing rehabilitative devices for disabled people. Our experiment confirmed that the proposed method outperformed the conventional methods implemented in the previous studies.

## 2. Literature Survey

Numerous rehabilitative devices have been developed in the past decade. Some of the important rehabilitative devices helping human welfare to overcome paralysis conditions were stated in the following. Y. Li et al. (2010) designed 2D cursor control for disabled people using an SVM classifier with common spatial pattern features and obtained an online classification accuracy of 97.5% from six subjects aged from 22 to 30 [22]. A. B. Usakli and S. Gurkan (2010) devised the virtual keyboard for people without hand movements and speaking using the Euclidean features with the nearest neighborhood algorithm and obtained classification accuracy of 95% [23]. A. Nanayakkara and Z. Sakkaff (2010) devised the EEG-based BCI using band power features with a KNN classifier and obtained accuracy of 85% from three normal subjects [24]. M. Phothisonothai and K. Watanabe (2013) designed an EEG-based application for disabled people from ten subjects using fractal dimension features with an ANN classifier and achieved classification accuracy of 83.25 [25].

H. H. Pang et al. (2014) developed an EEG-based controller for disabled individuals using Independent Component Analysis to classify the one-class imagery task using the proposed support vector data description classifier and obtained a high classification accuracy [26]. V. Gandhi et al. (2014) promoted a new EEG-based system for motor neuron affected people using band power features with RQNN and obtained accuracy of 89.00% compared to other classifiers used in the study [27]. A. Turnip et al. (2017) developed a wheelchair for spinal cord injured people using SSVEP-based featured signals trained with an adaptive network-based fuzzy algorithm and obtained classification accuracy of 90% [28]. A. R. Sereshkeh et al. (2017) designed a speech synthesizer for disabled people using autoregressive and discrete wavelet transform features trained with an SVM classifier and attained accuracy of 95.9% from 12 subjects [29].

O. R. Pinheiro et al. (2018) created a wheelchair using statistical features and a recurrent neural network classifier and obtained classification accuracy of 74.96% from 106 subjects [30]. X. Xiaoxiao et al. (2019) modeled EEG-based BCI for spinal cord injured people using CWT features trained with optimized NN classifiers and attained the accuracy of 97.50% in online mode for four tasks using a three-electrode system [31]. LiKai et al. (2019) designed an assistive device based on EEG signals for ALS-affected people using local binary patterns features trained with GWONN and obtained classification accuracy of 98.3% for male subjects, 95.00% for female subjects, and 88.33% for ALS-affected people [32]. M. K. Andrade et al. (2020) designed EEG-based BMI using continuous wavelet transform (CWT) features trained with multiple classifiers and obtained average classification accuracy of 99% for four tasks normally [33]. M. Thilagaraj et al. (2021) conducted a study to design a mobile robot using EOG signals from 20 subjects by implementing convolution theorem with distributed time delay and Elman neural network and achieved accuracy of 90.56% and 90.82% for classification and 83.24% and 83.55% for recognizing accuracy [34]. In this research work, we planned to study the performance of different age groups from 20 to 28 and 29 to 40 through an optimized neural network trained using a crow search algorithm to identify and select the best subjects for creating a global database for future online study. By identifying the best dataset, we can drive the mobile robot online without any training.

## 3. Proposed Methodology

**Figure 1:**
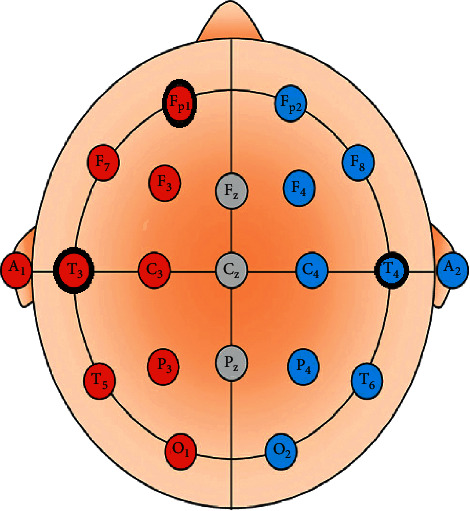
Electrode placement implemented in this study.

**Figure 2:**
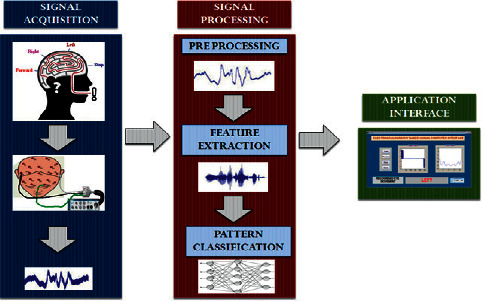
Methodology used in this research.

By placing three electrodes on the scalp of the forehead at the position of T3 and T4 (right and left) and ground electrode FP1 at the position above the left eyebrow, which is marked and depicted in Figure 1, participants were recommended to pronounce the four tasks constantly to record the data. The signals were composed for five seconds per trial. The recorded data were preprocessed using a notch filter with 50 HZ to remove the noise. The feature extraction method was applied to the preprocessed signals to identify the relevant information. Gained features were trained with an optimized NN classifier to identify the pattern, which is illustrated in Figure 2. The identified pattern was visualized with the help of the application interface. From the application interface control, commands were generated to control the external devices.

### 3.1. Experiment Setup

#### 3.1.1. Preliminary Study

**Figure 3:**
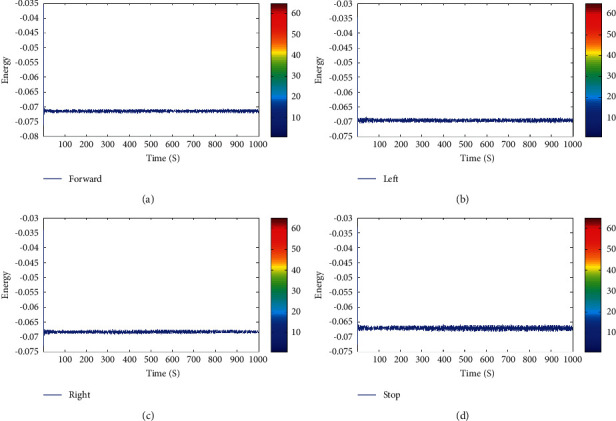
Collected raw EEG signals for the tasks (a) forward, (b) left, (c) right, and (d) stop in offline mode.

Before conducting this experiment, we conducted our preliminary study with five subjects to analyze the four signal patterns. Subjects who participated in the study evolved different patterns for each task. From the preliminary study, we identified the difficulties faced by the subjects while acquiring signals, as well as different patterns generated at the time of data collection, as shown in Figure 3.

#### 3.1.2. Protocol Design

All the participants must follow the protocol compulsorily and strictly without any deviation to collect the exact patterns to determine the tasks performed by them. After the preliminary study, we request all the participants to do the four tasks as per the protocol mentioned in the following.

*Right*. The subjects were informed to mentally assume the position RIGHT without vocalization, constantly for five seconds during data acquirement, and at the same time, subjects were requested not to make any obvious movement.

*Forward*. The subjects were asked to mentally constitute a letter FORWARD without vocalization constantly for five seconds during data acquirement, and concurrently, subjects were advised not to make any obvious movement.

*Left*. The subjects were instructed to mentally constitute a letter LEFT without vocalization constantly for five seconds during data acquirement, and at the same time, subjects were requested not to make any observable movement.

*Stop*. The subjects were instructed to mentally constitute a letter STOP without vocalization constantly for five seconds during data acquirement, and concurrently, subjects were advised not to make any overt movement.

All the volunteers involved in this study must follow the protocol strictly and seriously to generate good patterns at the time of signal collection.

#### 3.1.3. Signal Acquisition

Subjects were asked to sit comfortably on the chair in a relaxed position. During the subject selection, we found some difficulties. The parameters we applied to select the subjects are given as follows.

The subjects who participated in this study were aged between 20 and 40, and all were our university students and faculty members.

Twenty subjects were involved in this study. All the participants were right-handed and free from medical illness, and also they wore cotton clothes to feel free and comfortable.

The room was not covered with any soundproof materials. Participants were directed to pronounce the name of the tasks continuously as per the protocol for five seconds to acquire the signals.

At the time of acquisition, the signals were treated with a notch filter to take away the noises that affect EEG. Ten trials were executed per task. Forty data samples per subject were collected. EEG dataset used in this study was collected lively from the subjects. Collected signals were sampled at 200 Hz. A total of 800 samples from twenty subjects were collected to select the most related features.

### 3.2. Feature Extraction

Feature extraction was a technique to reduce the high dimensionality data into feasible data for processing and interpreting. Feature extraction methods were categorized into two types:

- Parametric method.
- Nonparametric method.

In our study, we focused on the parametric method to pick the quality features from observed preprocessed signals. Preprocessed signals were treated with the autoregressive Yule-Walker method to obtain the outstanding features from the observed data. The Yule-Walker technique was introduced by Udny Yule and Gilbert Walker. By combining both names, the name Yule-Walker comes. It was otherwise called an autocorrelation technique that is used to carry out the Power Spectral Density (PSD) from the input sample *b* fitting the windowed input data from the autoregressive model, and also it reduces the forward prediction error in the least square sense. So they are called AR Yule-Walker. It has the capacity to measure both stationary and nonstationary time series. It accepts the input in the form of a column vector. This column vector consists of the PSD of the specified trial signals. The frequency range of the signal was always between [0, *F*_*s*_], where *F*_*s*_ represents the sampling frequency of the signal [35, 36]. The mathematical representation for the AR Yule-Walker equation was represented in equation (1):

(1) $$\begin{array}{c} \gamma_{\text{m}}=\sum_{k=1}^{p}\varphi_{k}\gamma_{m-k}+\sigma_{\epsilon }^{2}\delta_{m,o}, \end{array}$$

where *γ*_*m*_ indicates the autocorrelation function, *σ*_*ϵ*_ represents the noise input's standard deviation, and *δ*_*m*,*o*_ illustrates the Kronecker delta function. If *m* = 0 indicates the nonzero values in the last part of the individual equation, the following equation can be solved by matrix format *b* representing the condition *m* > 0 [34, 35], which was represented in equation (2):

(2) $$\begin{array}{c} \left[\begin{array}{c} \gamma_{1}\\ \gamma_{2}\\ \gamma_{3}\\ \vdots \\ \gamma_{p} \end{array}\right]=\;\left[\begin{array}{c} \begin{array}{ccc} \gamma_{0} & \gamma_{-1} & \gamma_{-2}\\ \gamma_{1} & \gamma_{0} & \gamma_{-1}\\ \gamma_{2} & \gamma_{1} & \gamma_{0} \end{array}\\ \begin{array}{ccc} \vdots & \vdots & \vdots \end{array}\\ \begin{array}{ccc} \gamma_{p-1} & \gamma_{p-2} & \gamma_{p-3} \end{array} \end{array}\right]\left[\begin{array}{c} \varphi_{1}\\ \varphi_{2}\\ \varphi_{3}\\ \vdots \\ \varphi_{p} \end{array}\right]. \end{array}$$

Equation (2) can be solved from the condition {*φ*_*m*_ :  *m*=1,2,………….,*p*}, and the remaining equation for *m* = 0 so that

(3) $$\begin{array}{c} \gamma_{0}=\sum_{k=1}^{p}\varphi_{k}\gamma_{-k}+\sigma_{\epsilon }^{2}, \end{array}$$

where *γ*_1_, *γ*_2_,…*γ*_*p*_ represent the time series signals and *K* = 1, 2,…*p* indicates the order of the model. 1000 samples were given as input to the feature extraction technique, and 22 features were selected from each trial. Repeat the same steps for ten trials per task to develop the master dataset to classify the data offline and online using an optimized neural network.

## 4. Classification Technique

### 4.1. Feed Forward Neural Network (FFNN)

The benchmark network model in the artificial neural network (ANN) was called FFNN. It is otherwise called a static neural network. Because there was no feedback connection between the three layers, all the three layers were moved only in the frontward direction from the input layer to the hidden layer and from the hidden layer to the output layer. Basically, FFNN was trained with default parameters with a default training algorithm [37]. But in our research, we planned to change the benchmark training algorithm to a bioinspired optimization algorithm called crow search algorithm (CSA) to optimize the neural network model to analyze the different patterns generated from the subjects during the classification process, and also we compared the results gathered in the study.

### 4.2. Crow Search Algorithm (CSA)

CSA was one of the metaheuristic algorithms designed to solve several real-world problems compared with other conventional search methods, and also it provides the best results in complex design problems in the field of engineering. CSA was first introduced by A. Askarzadeh in 2016. This optimization algorithm imitates the basic behavior of crows in their social habit in terms of storing and retrieving excess food. Crow was one of the most intelligent birds. The intelligence of crows was dependent upon the size of their bodies. Crows have the capacity to recollect the images and dangerous places near their living area and warn the other crows through some sort of communication and also remember the food hiding places for a long time. This approach makes crows more clever, aware, and knowledgeable than other birds. Crows have the capacity to analyze the other birds' food hiding areas and steal the food from these places once the owner crow leaves the place. Crows are always behaving with extra precaution to save the food hiding places from crows [38].

#### 4.2.1. Working Principles of CSA

- Crows are always living in groups.
- During the time of searching for food, if any excess amount of food is found, the crow hides the excess amount of food in some other places for a long time and keeps the place clear like human beings.
- Crows have the capacity to watch other crows or birds' food areas to steal their food once the owner moves from the place.
- Crows take precautionary steps like humans to protect their own caches; if one of the crows follows the resident place, then immediately it starts to move to another place to fool the crow or other birds. Mathematical representations of the above-mentioned points are given in equations (1) and (2).

#### 4.2.2. Basic Concepts of CSA

Crows are always living in groups. Crows hide the food from other crows and remember the place. They have a tendency to save food in unknown areas for a long time and retrieve the saved food, remembering the hiding place very easily compared to other birds. If some crows are trying to follow the crow's unknown food hiding place, they immediately change the position and their original path in order to divert the following crow to save the hiding food. Let us assume that *N* is the number of crows in the group with *d* dimensional environment and let *i* be the position of crows in the search space, which can be represented by a vector [45–48], which was represented in equations (1):

(4) $$\begin{array}{c} X^{\text{iter}}=X_{1}^{\text{i,ter}}X_{2}^{\text{i,ter}}\ldots X_{d}^{\text{i,iter}},\\ X^{i,\text{iter}}=\left(1,2,\ldots \ldots \ldots .,\;N_{i}\right),\\ \text{iter}=\left(1,2,\ldots \ldots \ldots .,\;iter_{\text{max}}\right), \end{array}$$

where iter_max_ indicates the highest number of iterations.

Each crow in the flock has separate memory to memorize the hiding place of the food. Iter represents the iteration and hiding place of Crow_*i*_, and it was represented as *m*^*i*,iter^. From this equation, we can fix the best position of each crow from the search space environment. Suppose that Crow_*j*_ needs to visit the hiding place *m*^*j*,iter^ and Crow_*i*_ is planning to follow Crow_j_ to identify the hiding place of the food. This condition can occur in two ways.

**Condition 1 .**

Assume that Crow_*i*_ follows Crow_*j*_ but Crow_*j*_ does not know that Crow_*i*_ is following it. The mathematical representation for condition 1 was given in equation (4).

(5) $$\begin{array}{c} X^{i,\text{iter}+1}=X^{i,\text{iter}}+r_{i}xfl^{i,\text{iter}}x\left(m^{j,\text{iter}}-X^{i,\text{iter}}\right). \end{array}$$

From this equation, *r*_*i*_ represents the random number between 0 and 1 with uniform distribution, *I* indicates the iteration, and *fl*^*i*,iter^ specifies the crow flight length by unknowingly Crow_*i*_ following Crow_*j*_ to identify the food hiding place of Crow_*j*_. For this movement, the actual position of Crow_*i*_ was changed, and a new position was obtained, which was shown in equation (4).

**Condition 2 .**

If Crow_*j*_ identifies that Crow_*i*_ is following it, immediately Crow_*j*_ switch over from the old hiding place to the new hiding place to protect the food and to fool the Crow_*i*_ search position so that this term can be expressed mathematically by applying equation (5).

(6) $$\begin{array}{c} X^{i,\text{iter}+1}=\left\{\begin{array}{cc} X^{i,\text{iter}}+r_{i}xfl^{i,\text{iter}}x\left(m^{j,\text{iter}}-X^{i,\text{iter}}\right)r_{j} & \geq RP^{i,\text{iter}}\\ \text{arandomposition}, & \text{otherwise}. \end{array}\right) \end{array}$$

From this above-mentioned equation, *r*_*i*_ represents the random number between 0 and 1 with uniform distribution, *j* represents the number of iterations, and *RP*^*i*,iter^ indicates the recognizing probability of Crow_*j*_.

#### 4.2.3. Steps Involved in Optimization Algorithm

**Step 1 .**

Fix the problem and adaptable parameters. Fix all the adaptable parameters like the number of crows and their size (N), flight length (fl), recognizing probability (RP), and the number of maximum iterations (iter_max_).

**Step 2 .**

Fix position, search place, and memory of crow. In the beginning, the crow has no knowledge. So initially, crows have stored their food in an initial position. From this, each crow's position was fixed. The mathematical representation for fixing the memory of the crow and its positions is shown in equations (6) and (7).

(7) $$\begin{array}{c} \text{Memory}=\left[\begin{array}{c} \begin{array}{ccc} M_{1}^{1} & M_{2}^{1} & M_{d}^{1}\\ M_{1}^{2} & M_{2}^{2} & M_{d}^{2}\\ \cdots & \cdots & \cdots \end{array}\\ \begin{array}{ccc} M_{1}^{N} & M_{2}^{N} & M_{d}^{N} \end{array} \end{array}\right], \end{array}$$

(8) $$\begin{array}{c} \text{Crows}=\left[\begin{array}{c} \begin{array}{ccc} X_{1}^{1} & X_{2}^{1} & X_{d}^{1}\\ X_{1}^{2} & X_{2}^{2} & X_{d}^{2}\\ \cdots & \cdots & \cdots \end{array}\\ \begin{array}{ccc} X_{1}^{N} & X_{2}^{N} & X_{d}^{N} \end{array} \end{array}\right], \end{array}$$

where N indicates the random position in the search place and *d* indicates the decision variable.

**Step 3 .**

Calculate the fitness function. By implementing the decision variable into the fitness function to measure the position of each crow.

**Step 4 .**

Create a new position. Crow_*i*_ planned to discover the hidden food place of Crow_*j*_. For this, Crow_*i*_ randomly picks one of the crows from the groups and target the specified crow (*m*^*j*^). So the new position of Crow_*i*_ was obtained, which was shown in equation (5).

**Step 5 .**

Identify the possibilities of fixing reposition and checking the feasibility of the crow's reposition. If the new position was comfortable for the crow, then it changed its position; otherwise, the crow stayed in its previous own position and did not move to another position.

**Step 6 .**

Calculate the new position's fitness function. If the crow changed their new position for each and every move, the fitness function value of the crow was calculated.

**Step 7 .**

Restore the memory. Crows restore their memory, as shown in equation (8).

(9) $$\begin{array}{c} m^{i,\text{iter}+1}=\left\{\begin{array}{c} X^{i,\text{iter}+1\;f\left(X^{i,\text{iter}+1}\right)\text{isbetterthanf}\left(m^{i,\text{ iter}}\right)}\\ m^{i,\text{iter}}\text{Otherwise} \end{array}\right). \end{array}$$

If the crow's new position is superior to that of the fitness function of the previous memorized position, then the crow restores the memory value using the new position. In this equation, *f*(*m*^*i*,iter^) indicates the objective function value.

**Step 8 .**

Analyze the stopping criterion. Repeat steps from 4 to 7 until the maximum iteration iter_max_ is attained. The best position of the crow was met when the reposition of the crow was superior to the fitness function. The new best position was fixed as an objective function and restored the memory and optimization algorithm stopping its iteration [38–41]. After the training, the result of the study was discussed in the experimental study.

## 5. Experimental Study

The study was conducted with twenty subjects aged from 20 to 48 using the AR Yule-Walker features with crow search optimization-based FFNN to check the performances for developing the BCI for the disabled person.

### 5.1. Classification Accuracy

**Figure 4:**
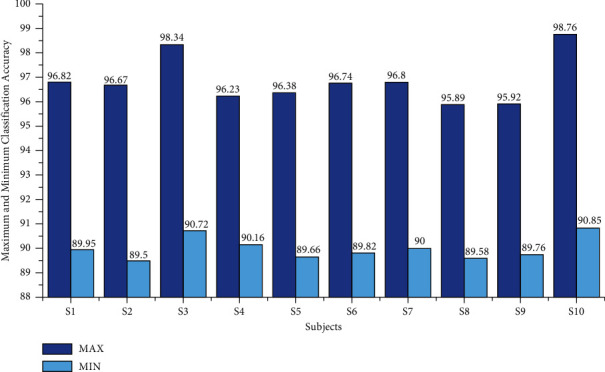
Maximum and minimum accuracy of subjects of the age group 20–28 using the AR Yule-Walker features with FFNNCSA.

**Figure 5:**
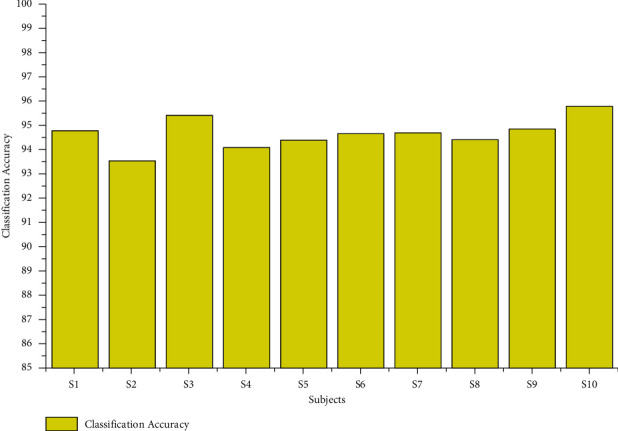
Overall classification accuracy of subjects of the age group 20–28 using the AR Yule-Walker features with FFNNCSA.

The classification accuracy for the age group between 20 and 28 using the AR Yule-Walker features with crow search optimization-based FFNN is depicted in Table 1. From Table 1, we determined that the average maximum and minimum classification accuracy were 96.86% and 90.00%, as shown in Figure 4. The overall classification accuracy of the age group between 20 and 28 was 94.66%, with an 18.56 sec training time and 0.74 sec testing time with standard deviation variations of 1.38 to 1.87. The individual maximum accuracy of 95.78 was attained for the subject S10, and the individual minimum accuracy of 93.54% was attained for the subject S2, as shown in Figure 5.

**Figure 6:**
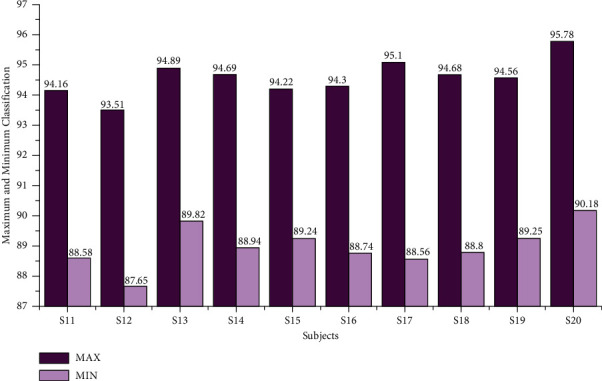
Maximum and minimum accuracy of subjects of the age group 29–40 using the AR Yule-Walker features with FFNNCSA.

**Figure 7:**
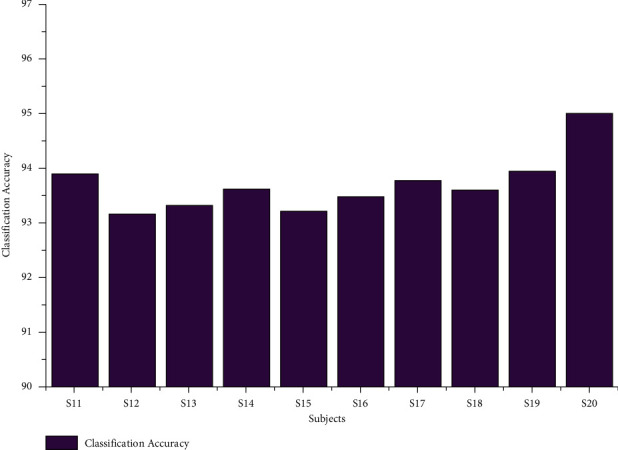
Overall classification accuracy of subjects of the age group 29–40 using the AR Yule-Walker features with FFNNCSA.

The classification accuracy for the age group between 29 and 40 using the AR Yule-Walker features with crow search optimization based FFNN is depicted in Table 2. From Table 2, we analyzed the average maximum and minimum classification accuracy of 95.00% and 89.41%, as shown in Figure 6. The overall classification accuracy of the age groups between 29 and 40 was 94.18%, with a 19.54 sec training time and 0.78 sec testing time with standard deviation variations of 1.62 to 1.77. The individual maximum accuracy of 95.00% was attained for the subject S20, and the individual minimum accuracy of 93.90% was attained for the subject S12, as shown in Figure 7.

**Figure 8:**
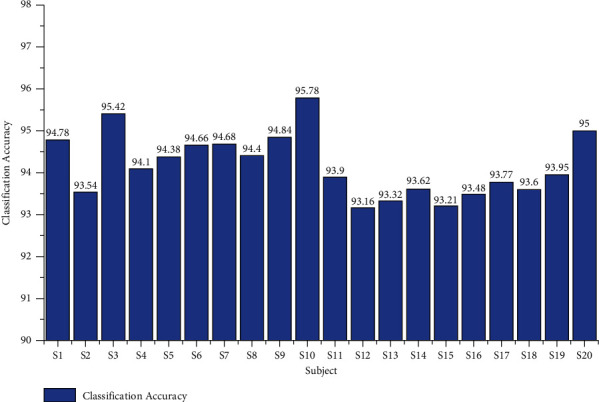
Overall accuracy of twenty subjects from the two different age groups 20–28 and 29–40 using the AR Yule-Walker features with FFNNCSA.

From Tables 1 and 2, we concluded the average classification performances of the two different age groups. The two tables proved that the average classification performances of the age group between 20 and 28 were appreciated compared to the group of 29–40, as shown in Figure 8. During the study, the performance of subjects belonging to the age group 20–29 was high at the time of data collection, and also they actively participated in the study. They were perfectly performing the tasks more efficiently than those of the age group 29–40.

### 5.2. Offline Test

**Figure 9:**
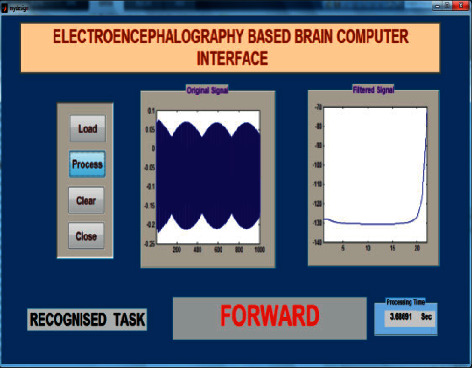
Task identification using GUI in offline mode for mentally composed task forward.

**Figure 10:**
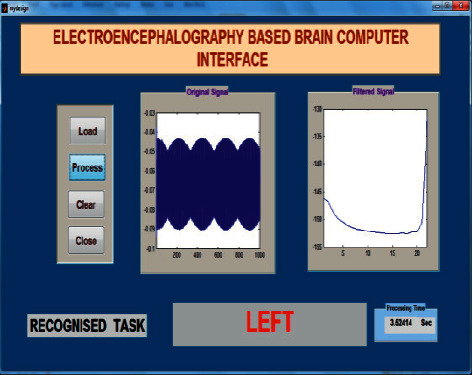
Task identification using GUI in offline mode for mentally composed task left.

**Figure 11:**
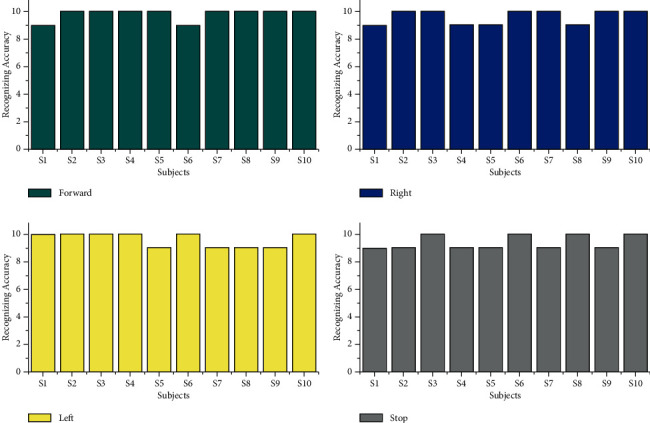
Taskwise recognizing accuracy in offline mode using STA for the age group 20–28.

**Figure 12:**
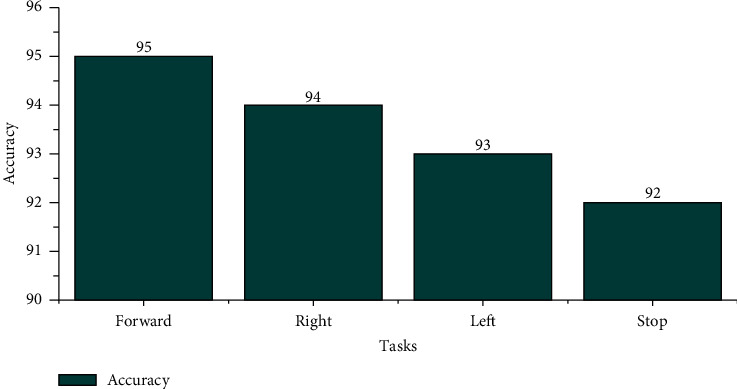
Taskwise offline recognizing accuracy using STA for the age group 20–28.

The offline test was conducted for both age groups 20–28 and 29–40 separately using the AR Yule-Walker features with crow search optimization based FFNN classifier. The offline task identification was conducted using GUI shown in Figures 9 and 10 to identify the mentally composed words. The offline recognizing accuracy for the age group 20–28 using Single Trail Analysis (STA) is displayed in Table 3. From Table 3, we pinpoint that the maximum offline recognizing accuracy of 100% was obtained for subjects S3 and S10 and the minimum offline recognizing accuracy of 92.50% was obtained for subjects S1 and S5. The individual single trial to identify the offline performances of the subjects belonging to the 20–28 age group is shown in Figure 11. Individual task accuracy of 98% for a forward task, 96% for right and left tasks, and 94% for stop task was obtained, as shown in Figure 12.

**Figure 13:**
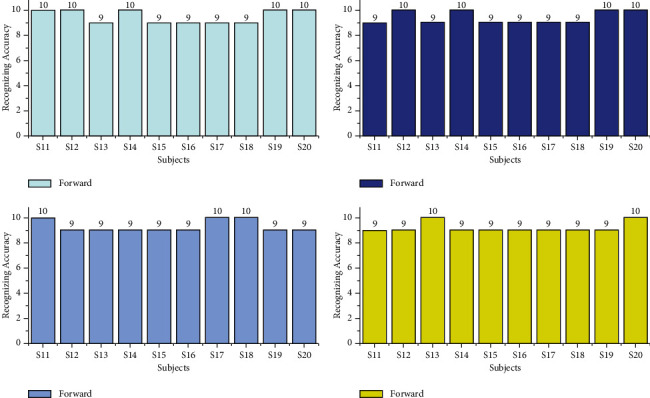
Taskwise recognizing accuracy in offline mode using STA for the age group 29–40.

**Figure 14:**
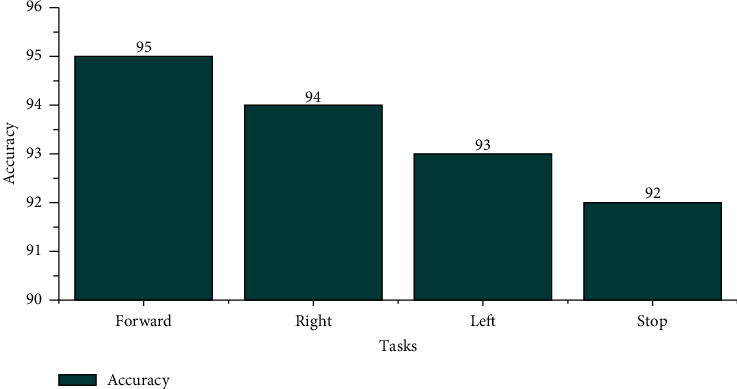
Taskwise offline recognizing accuracy using STA for the age group 29–40.

The offline recognizing accuracy using STA for the age group 29–40 is shown in Table 4. From Table 4, we discovered that the maximum offline recognizing accuracy of 100% was obtained for subject S20, and the minimum offline recognizing accuracy of 90.00% was obtained for subjects S15 and S16. The individual single trial to identify the offline performances of the subjects belonging to the 20–28 age groups is shown in Figure 13. Individual task accuracy of 95% for the forward task, 94% for the right task, 93% for the left task, and 92% for the stop task was obtained, as shown in Figure 14. From the analysis, we found that offline recognizing accuracy for the age group 20–28 was high compared to the age group of 29–40. From Tables 3 and Table 4, we concluded that, in both trialwise and individual taskwise, the recognizing accuracy of the age group 20–28 was high.

### 5.3. Online Test

**Figure 15:**
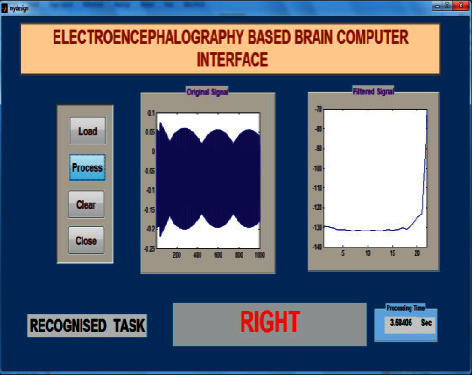
Task identification using GUI in online and offline mode for mentally composed task right.

**Figure 16:**
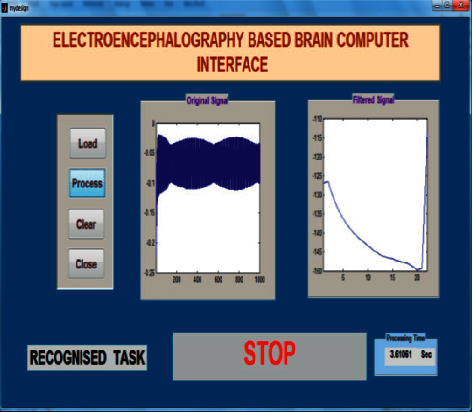
Task identification using GUI in online and offline mode for mentally composed task stop.

**Figure 17:**
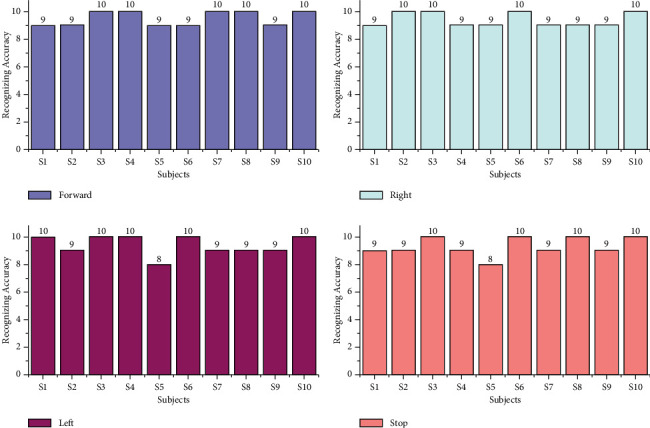
Taskwise online recognizing accuracy using STA for the age group 20–28.

**Figure 18:**
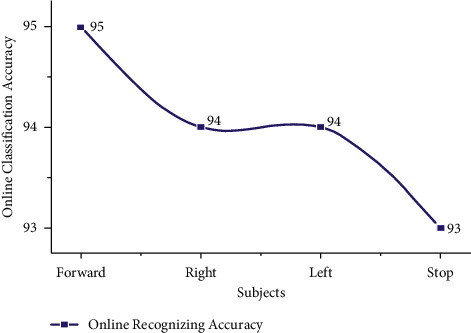
Taskwise online recognizing accuracy using STA for the age group 20–28.

The online task identification performances were conducted using GUI to classify the mentally composed words for two different age groups 20–28 and 29–40 using the AR Yule-Walker features with crow search optimization-based FFNN classifier. The online task identification performances were conducted using GUI illustrated in Figures 15 and 16 to classify the mentally composed words, and its recognizing accuracy performances were shown in Tables 5 and 6. From Table 5, the maximum accuracy of 100% was found for subjects S3 and S10 and the minimum online recognizing accuracy was found for subject S5. Individual trial performances of each subject are shown in Figure 17. Individual online task recognizing accuracy of 95% for the forward task, 94% for right and left tasks, and 93% for stop task is depicted in Figure 18.

**Figure 19:**
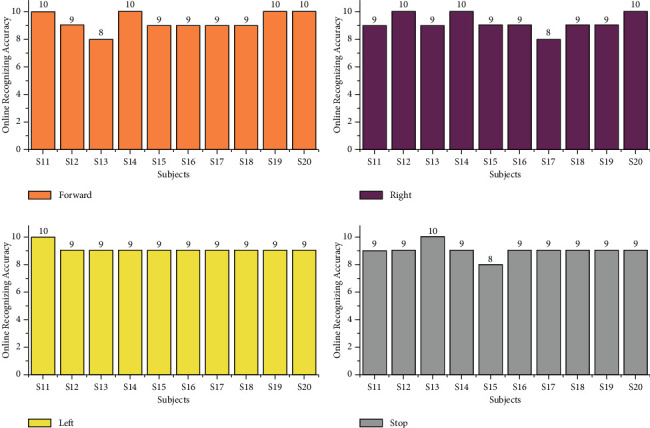
Taskwise online recognizing accuracy using STA for the age group 29–40.

From Table 6, we identified that the maximum accuracy of 95% for subjects S20 and minimum online recognizing accuracy of 87.50% was found for subjects S15 and S17. Individual trial performances of each subject belonging to the age groups of 29–40 are shown in Figure 19. Individual online task recognizing accuracy of 93% for the forward task, 92% for right and left tasks, and 91% for the stop task is depicted in Figure 18.

**Figure 20:**
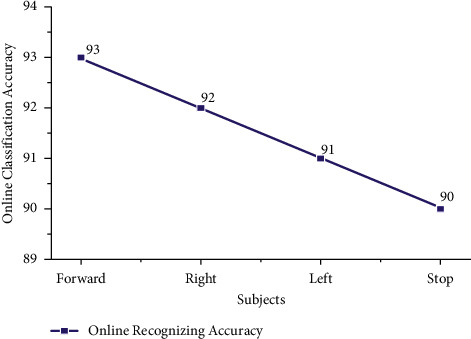
Taskwise online recognizing accuracy using STA for the age group 29–40.

From this offline study, our experiment has obtained the individual task accuracy of 98% and 95% for the forward task, then 96% and 94% for right and left tasks, and finally 94% and 92% for stop task were obtained for the age groups in range of 20–28 and 29–40 respectively. From the online study, we have obtained the recognition accuracy of 95% and 94% for forwards task, then 94% and 92% for right and left tasks, and finally 93% and 91% for the stop task for the age group in the range of 20–28 and 29–40 which were demonstrated in Figure 18 and Figure 20, respectively. Through this analysis, we concluded that subjects from the age group 20–28 concentrated effectively and performed the tasks comparatively higher and accurately compared with the age group 29–40.

## 6. Conclusion

In our study, we planned to analyze the best performances between two age groups 20–28 and 29–40 using the AR Yule-Walker features with crow search optimization-based FFNN classifier. The overall classification accuracy of the age group 20–28 was 94.66%, with an 18.56 sec training time and 0.74 sec testing time with standard deviation variations of 1.38 to 1.87. Through the offline test, we pinpoint that the maximum offline recognizing accuracy of 100% was obtained for subjects S3 and S10 and the minimum offline recognizing accuracy of 92.50% was obtained for subjects S1 and S5. Individual task accuracy of 98% for the forward task, 96% for right and left tasks, and 94% for the stop task was obtained. From the online recognizing accuracy, the maximum accuracy of 95% for forward task, 94% for right and left tasks, and 93% for stop tasks was obtained. Experimental results proved that subjects from the age group 20–28 concentrated effectively and performed the tasks comparatively higher and accurately compared with the age group 29–40.

## 7. Future Study

In our future study, we planned to conduct the online performances through the external devices to navigate and check the possibilities of designing the BCI in outdoor environment by fixing the obstacles detecting sensors to detect the obstacles present in front of the moving mobile robot with affected individuals.

## Figures and Tables

**Algorithm 1:**
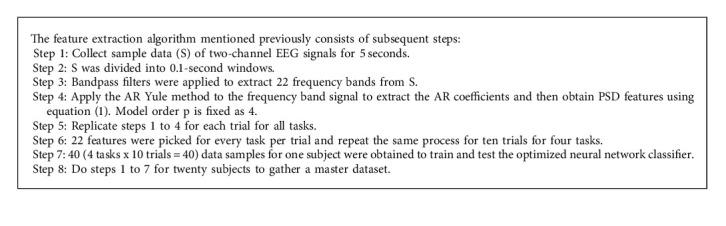
AR Yule-Walker method-based extraction.

**Table 1:** Performances of subjects of the age group 20–28 using the AR Yule-Walker features with FFNNCSA.

Subjects	Ten trials' average training time (sec)	Ten trials' average testing time (sec)	Average classification accuracy (%)			
			Sd	Max	Min	Mean
S1	18.79	0.76	1.87	96.82	89.95	94.78
S2	18.62	0.78	1.73	96.67	89.50	93.54
S3	18.30	0.72	1.38	98.34	90.72	95.42
S4	18.32	0.77	1.53	96.23	90.16	94.10
S5	18.95	0.73	1.68	96.38	89.66	94.38
S6	18.68	0.75	1.57	96.74	89.82	94.66
S7	18.44	0.76	1.62	96.80	90.00	94.68
S8	18.56	0.74	1.70	95.89	89.58	94.40
S9	18.78	0.72	1.71	95.92	89.76	94.84
S10	18.18	0.71	1.34	98.76	90.85	95.78

**Table 2:** Performances of subjects of the age group 29–40 using the AR Yule-Walker features with FFNNCSA.

Subjects	Average training time for ten trials (sec)	Average testing time for ten trials (sec)	Average classification performance (%)			
			Sd	Max	Min	Mean
S11	19.65	0.79	1.73	94.16	88.58	93.90
S12	19.74	0.78	1.70	93.51	87.65	93.16
S13	19.58	0.76	1.75	94.89	89.82	93.32
S14	19.42	0.77	1.77	94.69	88.94	93.62
S15	19.68	0.72	1.66	94.22	89.24	93.21
S16	19.36	0.73	1.69	94.30	88.74	93.48
S17	19.47	0.75	1.68	95.10	88.56	93.77
S18	19.74	0.81	1.73	94.68	88.80	93.60
S19	19.78	0.80	1.72	94.56	89.25	93.95
S20	19.12	0.74	1.62	95.78	90.18	95.00

**Table 3:** Offline recognizing accuracy for the age group 20–28 using the AR Yule-Walker features with FFNNCSA.

Subject	Recognizing accuracy for the age group 20–28 using the AR Yule-Walker features with FFNNCSA technique				
	Forward	Right	Left	Stop	Wrongly classified trials
S1	9	9	10	9	3
S2	10	10	10	9	1
S3	10	10	10	10	0
S4	10	9	10	9	2
S5	10	9	9	9	3
S6	9	10	10	10	1
S7	10	10	9	9	2
S8	10	9	9	10	2
S9	10	10	9	9	2
S10	10	10	10	10	0
Total	**98**	**96**	**96**	**94**	**16**

**Table 4:** Offline recognizing accuracy for the age group 29–40 using the AR Yule-Walker features with FFNNCSA.

Subject	Recognizing accuracy for the age group 20–28 using the AR Yule-Walker features with FFNNCSA technique				
	Forward	Right	Left	Stop	Wrongly classified trials
S11	10	9	10	9	2
S12	10	10	9	9	2
S13	9	9	9	10	3
S14	10	10	9	9	2
S15	9	9	9	9	4
S16	9	9	9	9	4
S17	9	9	10	9	3
S18	9	9	10	9	3
S19	10	10	9	9	2
S20	10	10	9	10	1
**Total**	**95**	**94**	**93**	**92**	**25**

**Table 5:** Online recognizing accuracy for the age group 20–28 using the AR Yule-Walker features with FFNNCSA.

Subject	Online recognizing accuracy for the age groups 20–28 using the AR Yule-Walker features with FFNNCSA				
	Forward	Right	Left	Stop	Wrongly classified trials
S1	9	9	10	9	3
S2	9	10	9	9	3
S3	10	10	10	10	0
S4	10	9	10	9	2
S5	9	9	8	8	6
S6	9	10	10	10	1
S7	10	9	9	9	3
S8	10	9	9	10	2
S9	9	9	9	9	4
S10	10	10	10	10	0
Total	**95**	**94**	**94**	**93**	**24**

**Table 6:** Online recognizing accuracy for the age group 29–40 using the AR Yule-Walker features with FFNNCSA.

Subject	Online recognizing accuracy for the age group 29–40 using the AR Yule-Walker features with FFNNCSA				
	Forward	Right	Left	Stop	Wrongly classified trials
S11	10	9	10	9	2
S12	9	10	9	9	3
S13	8	9	9	10	4
S14	10	10	9	9	2
S15	9	9	9	8	5
S16	9	9	9	9	4
S17	9	8	9	9	5
S18	9	9	9	9	4
S19	10	9	9	9	3
S20	10	10	9	9	2
**Total**	**93**	**92**	**91**	**90**	**34**

## Data Availability

The data used to support the findings of this study are available from the corresponding author upon request.
